# ‘*To be honest, women do everything*’: understanding roles of men and women in net care and repair in Southern Tanzania

**DOI:** 10.1186/s12936-018-2608-7

**Published:** 2018-12-07

**Authors:** Angel Dillip, Zawadi Mageni Mboma, George Greer, Lena M. Lorenz

**Affiliations:** 1 Ifakara Health Institute, Kiko Avenue, Mikocheni, P.O. Box 78373, Dar es Salaam, Tanzania; 2 Department of Disease Control, London School of Hygiene and Tropical Medicine, Keppel Street, London, WC1E 7HT UK; 3 USAID/US President’s Malaria Initiative Tanzania, Old Bagamoyo Road, Msasani, Dar es Salaam, Tanzania

**Keywords:** Gender roles, Men, Women, Mosquito net, Net care, Net repair, Malaria

## Abstract

**Background:**

In Tanzania, the roles of men and women are classified based on the local cultural context. While men are usually the breadwinners, women are traditionally responsible for most domestic chores. Particularly for malaria prevention, studies in Africa have revealed women as being responsible for daily up-keep of the net. Using social role theory, this study explored the role of men and women in net care and repair and gender-related motivation and barriers to net care and repair in Tanzania.

**Methods:**

The study was conducted in the two villages of Ruangwa district in Lindi Region. The study applied qualitative approaches and carried out in-depth interviews and focus group discussions with men, women, women with children under the age of five, and village key informants.

**Results:**

Mosquito nets were valued by all participants as a protection measure against mosquitoes. Study findings indicate that net care and repair falls under a woman’s daily household responsibilities. While men were said to assist in stitching damaged nets, washing dirty bed nets was regarded inappropriate for men and not traditionally accepted. Motivation for net care and repair was reported to come from both men and women; for a woman keeping the net clean defined a caring and responsible woman, while men indirectly promoted net washing when complaining about nets being dirty. Women reported that men could do everything that women do regarding net care and repair, but that it does not fit into societal norms.

**Conclusion:**

With increased globalization in Tanzania, more women are becoming part of the workforce, which may limit their full commitment to net care and repair activities, leading to increased net damage, malaria incidences and higher costs for malaria treatment. The National Malaria Control Programme should consider incorporating research-informed gender-transformative messages into their behaviour change communication on mosquito nets and work closely with trusted Community Health Workers to inform communities about the importance of sharing responsibilities in net care and repair. It is acknowledged that changing people’s behaviour and practices is a long process, which will require a deep cultural and political shift.

## Background

In Tanzania, like everywhere else, the roles of men and women are classified based on the local cultural context. Tanzanian society is largely patriarchal and in many communities, women are under the control of men and often accorded to a lower social status [1]. Gender roles have, therefore, been stereotyped as being masculine and feminine, which affects the division of labour and resources within the household [2, 3]. Following the impact of globalization and the country’s efforts in addressing gender inequalities, more women in urban and rural areas are becoming involved in economic activities and going out to work to earn money [4]. The current expectations of their roles at household level, however, remain the same: after work, women are expected to cook, fetch water and conduct all household chores as usual [5], but it is unclear for how much longer women can focus on both demands as carefully as required.

Several studies have looked at the gender role division in traditional households when it comes to general well-being of family members [6, 7]. In Tanzania, for example, women are considered responsible for all domestic duties ranging from cooking, collecting water, taking care of patients and serving men [5, 8]. Studies on treatment-seeking behaviour for children indicate that women are the first ones to recognize illness symptoms because they spend most of their time with the children. The husband becomes involved in treatment-seeking, when it needs to be sought outside the home, as it is usually him who pays for treatment [9, 10].

When it comes to household gender roles in disease prevention, particularly against malaria, women are more likely to use mosquito nets than men as they tend to share nets with their young children and are more vulnerable to the disease when they become pregnant [11–13]. A recent study in Kenya, found that male-headed households adopted more prevention measures, including mosquito net use, than female-headed households, potentially due to their higher purchasing power and increased access to health information and knowledge [14].

Long-lasting insecticidal nets (LLINs) are one of the most effective tools to reduce malaria morbidity and mortality [15, 16]. In addition to nets being used, they must also be maintained in good condition to avoid the development of holes and tears, which will render the net less useful against mosquito bites [17, 18] and lead to the discarding of nets [19–21]. Net maintenance entails activities that aim to prolong its durability, particularly those related to care and repair [22–24]. As per World Health Organization definition, bed nets are designed to retain satisfactory amounts of insecticide to last for up to 20 washes and survive up to 3 years [24]. While caring is defined as washing, drying, proper hanging, careful tucking and untucking from underneath the mattress and net storage, net repair encompasses stitching holes with needle and thread, knotting or patching [22–24]. Studies indicate that women are primarily responsible for the daily up-keep of the nets, including washing and stitching when damage occurs [22–24]. In Uganda and Nigeria, men were reported to take part in repair to some extent, but not caring for nets [23, 24]. No published studies in Tanzania have looked at household roles in net maintenance.

Tanzania’s National Malaria Control Programme (NMCP) behaviour change communication (BCC) strategy focuses on the value of nets, the importance of sharing nets with others, the appropriate use of nets, careful handling of nets and methods of net repair [25], but there is still an important gap between the messages and people’s actions [26]. Understanding household dynamics and gender roles in net care and repair may inform appropriate interventions geared towards addressing gender-related challenges that currently inhibit net care and repair with the overall aim of increasing the life span of mosquito nets.

This study investigates the role gender plays in net care and repair behaviours in southern Tanzania through the lens of social role theory. Social role theory argues that household distribution of activities is based on societal expectations and stereotypes that are socially constructed, thus producing gender roles [2]. Such roles have been the main source of discrimination, which have been accepted by society at large. Eagly [2] divides gender roles into *communal* and *agentic*. The communal role is characterized by attributes of emotional and physical nourishment, commonly associated with domestic activities, and ascribed to women more than men. The agentic role, on the other hand, is characterized by features of confidence and forceful behaviour in public activities and is more likely to be associated with men.

Thus, the study aims to explore the roles of men and women in net care and repair activities at the household level in the context of perceived malaria risk and benefit of bed net use. The theory guided us in exploring gender-related motivation and obstacles to net care and repair; and gender-associated decisions in care and repair.

This study took place in the two villages of Southern Tanzania, which are part of the School Net Programme (SNP), a continuous distribution mechanism that uses school-going children as a means for delivering nets into the community [27]. The findings from the study aim to help the NMCP BCC to come up with relevant gender-related care and repair messages for men and women to be targeted more effectively.

## Methods

### Study area

The study was conducted in two villages in Ruangwa district (Lindi region, southern Tanzania) where SNP has been ongoing annually since 2013 [27]. Malaria prevalence in children aged 6–59 months in the Lindi region was 17.4% according to the 2015–2016 Tanzania Demographic and Health Survey and Malaria Indicator Survey [28]. The study villages were randomly selected from the Sample Vital registration with Verbal Autopsy (SAVVY) database [29]. SAVVY had randomly selected 15 villages within Ruangwa using probability proportional to size (PPS) sampling. For this study, one rural (Makanjiro) and one semi-urban (Kilimahewa) village was randomly selected using the ‘sample’ command in STATA14.

### Study design and participant selection

Focus Group Discussions (FGDs) and In-Depth Interviews (IDIs) were used to collect information from study participants. Interview methods took an inductive approach that allowed participants to report issues related to household roles in net care and repair while probing for necessary information [30]. The study participants were purposively selected with assistance from village leaders to ensure that relevant information is obtained necessary to answer the study objectives and capture differences in responses among the study groups. The sample size was determined using a combination of saturation sampling [31, 32] and reviewing similar studies [22–24].

In each village, a total of five FGDs was carried out; four with community members (young men (18–24 years old), women with children under the age of five (18+ years), older men (> 25 years), older women with or without children (> 25 years old)), and one FGD with village key informants (village, religious and traditional leaders and influential people aged 18 and above). The number of FGD participants ranged between 8 and 12 participants per group. In each village, 15 IDIs were conducted. The IDIs consisted of five men, five women with or without children, and five women with children under the age of five. In each village, response saturation was reached after three FGDs and five IDIs, but sampling was continued to ensure no more new themes emerged. Participants had to fulfil the following inclusion criteria: resident in study site for a minimum of 12 months, at least 18 years of age and owner of at least one insecticide-treated net (ITN).

### Data collection procedures

Prior to data collection, the study team carried out a pilot exercise in Pemba Mnazi, a rural village in Dar es Salaam region. One FGD and four IDIs were conducted with purposively selected residents to pilot the topic guides to check if they were locally appropriate. Based on the pilot study, the FGD and IDI guides were revised. FGDs were conducted at village offices while IDI participants were visited in their homes. All interviews and FGDs were conducted in Kiswahili language. The senior social scientist participating in the study conducted quality check of the IDIs by revisiting some of the households. Audio-recording devices were used while research assistants also took notes during each interview. All recorded interviews were transcribed.

### Data management and analysis

NVivo 11 Pro software was used for data management. Transcripts were coded and the list of codes were reviewed and grouped into categories and themes for analysis. From the codes, patterns and themes in the data were identified that answered the specific study objectives. Analysis was undertaken by comparing themes that answered key issues related to the study objectives and checking for inconsistencies across different data sources. After analysis, data from the two study villages, and IDIs and FGDS, were combined because of the similarity of the findings.

### Ethical considerations

Ethical clearance was sought from Ifakara Health Institute, and the Tanzanian National Institute for Medical Research (NIMR). Local authorities where the study took place were also informed. An information sheet about the study was drawn up in Kiswahili, explaining the study rationale and participant’s rights. Written consent was obtained from participants and a thumb print for those who could not write. Measures were taken to ensure privacy, respect and dignity of all participants. Identities of participants in the FGDs and IDIs remain anonymous.

## Results

### Perceived risks of malaria among men and women

Most of the study participants were both net owners and users. Study participants primarily used mosquito nets as a preventive measure against malaria. Mosquito nets were valued by participants in both villages as malaria was perceived to be a dangerous disease associated with economic and health risks. All participants perceived two distinct groups at the highest risk of malaria: (1) children under the age of 5 and (2) adults. Most women also said that pregnant women and their unborn babies were at higher risk than other groups. Both men and women see malaria as a disease leading to poverty: costs associated with treatment, sickness and death were their main concern. In addition, men also worried about their ability to perform their daily activities and feed the family when infected with malaria; once a man, usually the head of household, falls sick, the whole family will be in trouble as he will not be able to feed the family or pay for his children’s school fees.

“*Malaria is not a joke, you will be in bed for more than a week, joint pain, no energy, while you are supposed to work and fight so that the family survives*.” (Male IDI participant, Makanjiro)

### Family roles in net care

Net care was defined as keeping nets clean and tidy by washing, drying and hanging nets back over sleeping spaces after drying. In addition, daily net maintenance behaviours, such as careful tucking and untucking from underneath the mattress after a night’s use, and tying nets up during the day, were mentioned. Net care in the household was perceived to be the responsibility of women, usually the wife. This was confirmed by all male and female participants. Women were said to be responsible because they mainly remain at home taking care of the family when men go out to work. The roles and responsibilities of working women remained the same inside the house, including net up-keep.

“*The woman is the one who is more responsible to look after bed nets, she manages the house. As for me, I have to go and work to feed the family*” (Male FGD participant, Kilimahewa)

“*Women know when the net is dirty and needs to be washed, they are involved in daily up*-*keep of the net, men can only remind you to wash the net*” (Female FGD participant, Kilimahewa)

Looking further into the roles of men in daily net care, participants reported that men could only assist in “hanging the net after washing” and “tucking and untucking from under the mattress”. However, even these activities were said to be optional.

“*To be honest, women do everything, as for us men, majority of us wake up like 5 am in the morning and come back in the evening, we, however, somehow assist our wives in hanging a net when it is dried*” (Male Key Informant FGD, Makanjiro)

“*Maybe when I ask him to assist and only if he agrees, he can hang it back on the hanger*” (Female IDI participant, Kilimahewa)

However, most participants in both study villages reported that in situations where women were not available or travelling, men do take care of the nets, particularly hanging, tucking and untucking from under the mattress but not washing. Net washing was considered inappropriate for men and not traditionally accepted.

“*They do not wash net, oho, if people see your husband washing net, they would think you have bewitched him, people will also think that you have control over your husband*” (Female FGD participant, Makanjiro)

### Family roles in net repair

Net repair was defined as stitching holes with needle and thread, whereas knotting was described only as a temporary repair measure awaiting stitching in the coming few days. As with net care, most male and female participants reported net repair to be a female chore because women are the ones most likely to identify a hole during the daily net up-keep. However, women also acknowledged that men do assist in stitching holes. Male participants also reported to help their wives stitch nets whenever they identified a hole big enough to allow mosquitoes to enter the net.

“*It is us women who stitch, most of the time it is us, yes men do assist when they have time, they stitch*” (Female IDI participant, Kilimahewa)

Probing on why men were more willing to stitch than wash a net and the common theme was that net repair can be done more privately than net care. A man helping with net repair is more common than helping with net care possibly because net repair can be performed inside the house unlike net care, an activity performed outside the house.

“*In fenced houses, men can stitch a net, but with our environment people can pass anytime and see a man washing, so they stitch inside the house because no one will see them*” (Male Key Informant FGD, Kilimahewa)

This was also supported in IDIs: “*Men help us to stitch but not wash nets, if they wash it means they have to take it to the rope and dry it outside, people will see them, but stitching, they can do it inside the house*” (Female IDI participant, Makanjiro)

Related to the study objectives, the role of children was investigated in their engagement in all activities related to net care and repair as most of the nets within households had been obtained through the School Net programme. The responsibilities of children were said to depend on the age of the child. Starting from about age 13, some children were said to assist their parent in washing and stitching holes.

### Gender-related motivators and barriers to net care and repair

Motivation to care for and repair the damaged net was reported to come from both the husband and wife in the household. The study noted that women respondents were more interested in washing and keeping the net clean than stitching holes. Keeping the net clean was considered a good practice that defines a caring and responsible woman.

“*Yes, we wash our nets, when your husband wants to sleep and finds the net dirty and dusty, he complains, and it will look like you do not properly manage your duties*” (Female IDI participant, Makanjiro)

The same was reported by men during their FGD: for things to run smoothly in the household, the man has the say, and men remind their wives to keep nets clean and free from dirt to avoid other health problems such as respiratory infections.

Being over-occupied with household tasks was mentioned as the main reason for women not remembering to repair nets. Others reported their own ‘laziness’ as a contributing factor to not repairing mosquito nets. Women were of the view that it is more convenient to wash nets than to repair them because washing is already part of their daily household routine. They must wash their husband’s clothes and children’s school uniforms; in doing so, it is easy to also remember washing the mosquito nets. Stitching clothes, on the other hand, is done much less frequently.

“*Washing can be easier and more convenient than stitching, when you wash family clothes it is easy to remember that a net is dirty and wash it too, but with stitching, you know, it is not done everyday, you see the hole on your net and say, I will stitch later, later becomes later, and it is already a new day*” (Female FGD participant, Kilimahewa)

Women revealed that the cost of repairing a net is very small, involving 200 Tanzanian Shilling (0.10 US$) to buy a needle and thread which can be used for many years. Most study participants reported they did not take their damaged nets to a tailor. Taking a net to be repaired by a tailor was regarded as awkward, as a bed net is considered a private item that needs to be repaired within the household. Moreover, for a woman to take a net to be repaired by the tailor was considered irresponsible and shameful.

“*You know bed net is something private, not everyone should see your net, it should be stitched inside, how can a woman take her net to the tailor, that’s shame, big shame, if you cannot stitch your net, what can you do, you better leave it with holes than taking it to the tailor*” (Female FGD participant, Makanjiro)

Gender of the main income earner was said not to affect responsibilities and choices when it comes to net care and repair. Even when a woman is the one working and earning money for the family, she remains responsible for household activities including net care and repair. Most male and female participants did report that men have the ability to do everything when it comes to net care and repair (washing, hanging, tucking and untucking from under the mattress, stitching holes, etc.), but that they would be perceived differently by other village members if they performed these household duties regularly.

“*Sure, they can do everything, they can wash net, hang it, tuck it, there is nothing that they can’t do, it is just that it is not within our norm. Those are regarded as women’s responsibilities*” (Female FGD participant, Kilimahewa)

“*Yes, we can wash and stitch, but you know those are women duties, we are busy looking for money*” (Male FGD participant, Kilimahewa).

“*You make me laugh, even if she is the breadwinner and I have no job, I cannot perform those tasks, unless I choose to help her, those are her duties*” (Male FGD participant, Makanjiro)

## Discussion

In most traditional African societies, the role of women within the household are rooted in culture, laws and social norms [33]. Study findings indicate that net care and repair in this area of Tanzania falls under a woman’s daily household responsibilities like in other sub-Saharan African countries [22–24]. In contrast to men, women tend to spend more of their time at home while taking care of all household duties. Even in cases where women also work and leave early in the morning, or are the main income earner for the family, their role in net care and repair remains the same. Despite the recent employment transition where more women have become employed in traditionally male-dominated sectors, the average hours women work on domestic chores vastly exceeds that of men [34]. An in-depth study of women in Tanzania showed that women were overwhelmed with household duties, but that even after long days on the farm, a woman would still cook, collect water and perform other household-related duties [35]. Women in this study reported that they were often too busy to repair nets, leading to low net repair rates [36]. Thus, an important measure to protect against mosquito bites and malaria transmission falls by the way side due to the increasing demands of women—an issue that needs to be addressed by NMCPs.

Feinstein [5] argues that culture is an integral part of people’s life and changing such an important part of society is very difficult. In the Tanzanian context, women are brought up to do household duties like washing clothes, cleaning and cooking. If a man is found performing a woman’s duty, he is diverging from social norms and acting against the local culture. Study results fall along the lines of cultural expectations: men support and report to perform those net care and repair activities that are restricted to inside the house (e.g. hanging nets after drying, stitching holes) when their wives are not around, but they would not perform net-related household chores in view of others, e.g. washing of nets [23]. On the outside, people can see him and perceive him differently as he is acting against societal norms [35]. Additionally, many tailors in Tanzania are men and thus stitching is less considered a ‘woman’s’ activity and is more acceptable within society.

In most traditional African societies, men are still the main decision makers for family matters at large [37]. Interestingly, women report that men are able to do everything that women do regarding net care and repair, and that men prompt women to maintain the nets, particularly when they go to bed and realize that the net is not clean. Women know that men and women are the same and what a woman can do, a man can also do, but to put this knowledge into action, good communication between a husband and wife is required [5]. Gender-related interventions could work better among male-headed households where motivation to net care and repair comes from men. However, there is no published data showing that involving men in net care-related duties would lengthen the lifespan of nets or decrease the vulnerability to malaria. This will need to be studied in the future.

The findings from this study reflect what is argued in the social role theory [2]. There is a clear difference between men and women when it comes to household chores, in this case net maintenance activities. This is something that has been accepted by the study communities. Once one deviates from what is expected of her/him, it is regarded as abnormal. In Tanzania, like other developing countries, the social position of women exposes them to bear a higher portion of the work than men while being deprived of resources and decision-making power. While gender roles did not seem to hinder net care and repair activities in this study, it is important to note that, with increased globalization, women in Tanzania are becoming an ever-increasing part of the workforce [38]. This may limit their full involvement in domestic chores including those related to net care and repair, which, in turn, may lead to increased net damage and malaria attacks in the family. A study in Tanzania revealed how women are overwhelmed with both farming and domestic chores [35]. Since women acknowledge that men can also do everything that women do with regard to net care and repair and study findings reveal that men do take part in repair activities, it is important that programmes sensitize the involvement of men in care and repair activities for the health benefits of the family.

This is the first investigation in Tanzania into behaviours around LLINs through a “gender-aware” lens [39]. Understanding gender culture and attitudes towards net care and repair in this local context will allow NMCPs to create programmes that aim to transform ingrained gender norms rather than reinforce existing stereotypes (e.g. the man pays for treatment and the woman nurses the sick) or ignore differences based on gender. The current NMCP BCC materials do not include specific gender-related information on net care and repair but their pictures solely focus on women interacting with mosquito nets on behalf of their families. According to the Gender Equality Continuum Tool [39], to be truly gender-transformative and create equal and enabling environments, positive norms needs to be strengthened. Changing people’s behaviour and practices is a long journey and will require a deep cultural and political shift, effected by research-informed and situationally-tailored BCC interventions. Community programmes should highlight the burden women and men will face when they or their child catches malaria: loss of monetary income, paying for treatment which will make household economy fluctuate, longer-term sickness and potential death. Thus, it is the responsibility of the whole family to avert the dangers of malaria, for example by maintaining clean and intact mosquito nets. Men are already privately performing net care and repair activities. Reframing net care and repair from a household chore to something that protects the economic stability of the households may allow men to start exhibiting such behaviours more publicly. At the same time, women should be encouraged to ask for help and support from their children (both boys and girls) and their husbands, so that social norms are rebuilt from within households.

While this transition to equality is taking place, it is important that women are empowered by programs to conduct high quality net care and repair. Learning from Donner et al. [40], strategies to involve women in indoor residual spraying (IRS) activities in some African countries significantly increased the number of women employed in the programme. This ensured safety for women in their working place, encouraged women to apply for supervisory roles and guaranteed security of women during pregnancy. Women should receive more support to make net care and repair a priority for the benefit of their family and society at large.

## Conclusions

This study provides an in-depth look at household roles in net care and repair behaviours in southern Tanzania. While findings are consistent with what is reported elsewhere in Africa and other developing countries, this is the first study in Tanzania to investigate gender roles in net care and repair attitudes and actions. Currently, net care and repair activities fall under a woman’s domain of household chores while men choose to assist when and how they want (mostly repairing nets behind closed doors). As an effect of globalization, women in Tanzania are slowly becoming part of the work force. This may limit their involvement in household chores including those related to net care and repair, leading to increased net damage, frequent malaria incidences and higher costs for malaria treatment. Since men are already participating in repair activities, the NMCP should consider incorporating research-informed gender-transformative messages into their BCC activities on mosquito nets to reduce gender-related barriers to net care and repair. The BCC promotion should focus on the importance that men, women and children take responsibility for the upkeep of their mosquito nets and develop positive norms for men to perform maintenance activities not just inside the privacy of their own homes, but also publicly assisting each other in case the spouse is occupied with other tasks. The NMCP would benefit from working closely with Community Health Workers, because they are well trusted and may be good agents to inform communities about the importance of sharing responsibilities in net care and repair. The gender-inclusive messaging aims to enhance current maintenance practices to prolong net durability.

## Abbreviations

LLINs: long-lasting insecticidal nets
BCC: behaviour change communication
NMCP: National Malaria Control Programme
SNP: School Net Programme
SAVVY: Sample Vital Registraion with Verbal Autopsy
ITN: insecticide-treated net
NIMR: National Institute for Medical Research
IRS: indoor residual spraying
